# Chronobiology of transient global amnesia

**DOI:** 10.1007/s00415-021-10639-x

**Published:** 2021-06-08

**Authors:** Carolin Hoyer, Kyoko Higashida, Fabio Fabbian, Alfredo De Giorgi, Vesile Sandikci, Anne Ebert, Michael Platten, Shuhei Okazaki, Roberto Manfredini, Kristina Szabo

**Affiliations:** 1 Department of Neurology, Medical Faculty Mannheim and Mannheim Center for Translational Neurosciences, University Medical Centre Mannheim, Heidelberg University, Theodor-Kutzer-Ufer 1-3, 68135 Mannheim, Germany; 2 Department of Neurology, Osaka University Graduate School of Medicine, Osaka, Japan; 3 Department of Medical Sciences, University of Ferrara, Ferrara, Italy

**Keywords:** Transient global amnesia, Chronobiology, Circadian rhythm, Infradian rhythm, Season, Day-of-week

## Abstract

**Introduction:**

The etiology of transient global amnesia (TGA) is still a matter of debate. Based, among others, on the observation of a close temporal relation between certain events and subsequent TGA episodes, recent proposals discuss the relevance of stress-associated processes impacting on hippocampal functioning. Circadian, infra- and ultradian rhythmicity has been found to play a relevant role in the multifactorial pathomechanisms of various disorders but has not been thoroughly studied in TGA.

**Methods:**

Data of patients with a final diagnosis of TGA were collected in Mannheim, Germany (06/1999–01/2018, *n* = 404), and in the Kansai district, Japan (04/2006–03/2018, *n* = 261). Chronological patterns of TGA occurrence were determined.

**Results:**

Significant circadian rhythmicity of TGA occurrence with bimodal peaks (mid-morning, late afternoon) was found for the entire population (*p* = 0.002) and for either sub-cohort (Mannheim: *p* = 0.003, Kansai: *p* = 0.007). This finding was confirmed for either sex (women: *p* = 0.004, men: *p* = 0.004) and different age groups (< 65 years: *p* = 0.0009, ≥ 65 years: *p* = 0.003). There was no variation according to day of the week, month or season, but the proportion of patients with a weekday episode was significantly higher in the Mannheim cohort (*p* = 0.002).

**Discussion:**

We identified a robust circadian rhythm in TGA occurrence which remarkably applied to either of the two study sites located on different continents and which was independent of sex and age. In light of abundant evidence of circadian rhythmicity of both, components of the human stress response system and memory, chronobiological analyses may provide an opportunity to further uncover the mechanisms underlying TGA.

## Introduction

Transient global amnesia (TGA) is a neurological disorder characterized by an acute onset of dense anterograde amnesia, which is often accompanied by retrograde amnesia of varying extent, but an otherwise normal neurological status. Despite decades of intense investigation, the etiology and pathomechanism of the disorder remain enigmatic. Various theories have been proposed, none of which has been able to provide satisfactory explanations for the empirically observed epidemiological and clinical characteristics of TGA as well as account for the surprisingly low rate of recurrence in light of the rather common nature of precipitating circumstances. A recent systematic review on the role and relevance of conventional vascular risk factors in TGA patients found distinct differences in the risk profile of this patient population and patients with a diagnosis of transient ischemic attack [1], thus supporting an etiological separation of TGA from arterial ischemic disease. Regarding a comparison of TGA patients and healthy controls, the authors found that severe hypertension appears to be associated with TGA while diabetes mellitus may confer a protective effect.

The long-term risk of ischemic stroke after TGA is not elevated [2, 3], further arguing against the ischemic hypothesis of TGA etiology. Compromised venous hemodynamics has also been discussed as a possible mechanism underlying TGA but convincing evidence is lacking [4]. Patients with TGA exhibit higher prevalence of depressive symptoms, psychiatric comorbidities and [5, 6]—all common features of migraine [7] with which TGA shares some commonalities [8, 9]. These findings and the observation of a striking temporal association of attacks of TGA with certain types of events such as exercise, emotionally charged situations, or cold water immersion [6, 10], have contributed to a conceptualization of the syndrome as a stress-induced condition related to glutamate-mediated hyperexcitability with consecutive cytotoxic damage of hippocampus neurons, as well as a glucocorticoid-mediated impact of stress-associated hypothalamus–pituitary–adrenal (HPA) axis activation on hippocampal functioning [11–13].

The circadian clock is a program found in all living organisms, which confers a universal 24-h structure on a wide and diverse range of biological processes of varying complexity [14]. It generates an approximate 24-h rhythm even in the absence of zeitgebers such as the light–dark cycle but it is also impacted on by physical exercise as well as social environments and their requirements in animals and humans [15]. Daily fluctuations in the levels of demand and stress must be anticipated and adapted to maintain a certain degree of overall homeostasis. To this end, both the regulatory mechanisms themselves as well as the extent of responsiveness of target structures on different organizational levels display varying degrees of circadian oscillations [16]. Accordingly, apart from the primary clock in the suprachiasmatic nucleus of the hypothalamus, there is a range of peripheral clocks in almost all types of tissue [17–19]. In addition to circadian rhythms, there exist also considerably shorter and longer rhythms, and numerous ultradian rhythms have been identified in humans [20]. Seasonality of physiological processes, however, is less pronounced than in other species [21].

Investigating patterns of temporal relationships between occurrences of disease manifestation as well as within scopes of pre-existing external and internal rhythms may provide relevant information for advancing knowledge about the pathogenesis of disease [22]. Not surprisingly, then, many disorders have been looked at from this perspective. For example, circadian and seasonal variations of onset of acute (cardio-)vascular events such as myocardial infarction, dissection or rupture of aortic aneurysms and stroke [23–26] have been found to display circadian as well as ultradian variations of onset, and these insights have been supplementing and stimulating further research into, first, the interplay of predisposing and triggering factors within multifactorial etiopathological frameworks, and second, therapeutic approaches utilizing insights from chronobiology [27, 28]. Analogously, the objective of the present study was to determine whether TGA occurrence in two large cohorts of TGA patients exhibits circadian, weekly, and monthly or seasonal variations.

## Methods

### Study design and sample

Data of patients with a final diagnosis of TGA were collected from a prospectively collected database of patients consecutively admitted to the University Medical Centre, Mannheim, Germany, between June 1999 and January 2018 (*n* = 404) and the diagnosis made in accordance with the criteria by Hodges and Warlow [29] by trained neurologists. In line with these criteria, TGA was only assumed if there was no concurrent neurological or mental disorder, if the patient’s neurological examination was normal regarding cranial nerve, motor, sensory, and cerebellar functions, and if there were no signs indicating stroke or seizure in the context of presentation. In addition, patients with imaging findings not compatible with TGA were not included in the study.

Furthermore, patients were retrospectively selected from a database containing medical information of patients from four tertiary medical centers in two cities in the Kansai district of Japan [Osaka University Hospital (Osaka), the National Cerebral and Cardiovascular Center (Osaka), the National Hospital Organization Osaka National Hospital (Osaka), and Kobe City Medical Center General Hospital (Hyogo)] between April 2006 and March 2018 (*n* = 261). Patients were identified via the ICD-10 code for TGA, and medical records of all potential TGA patients were subsequently reviewed in detail by a trained neurologist who made the final diagnosis according to aforementioned criteria. Data analyzed included basic demographic information and time of symptom onset.

### Statistical analysis

Distributions of continuous variables between groups were compared with Student’s *t* test for independent samples, and distributions of categorial variables were compared using χ^2^ test. Statistical analysis was performed with IBM SPSS Statistics, version 27.

For patients with known time of onset of the TGA episode, the hour of each event occurrence was tabulated, and the event frequency for each hour of the day was calculated. Partial Fourier analysis to the time series data using Cosinor software (Time Series Analysis—Cosinor, Esvres, France) was performed for testing chronobiological pattern. The Cosinor method allows to identify rhythmic patterns, i.e., reproducible for each considered period, and the software permits the selection of the harmonic, or combination thereof, that best explain the variance of data. Acrophase (peak time of rhythmic change) and peak and trough times of the overall best fitted curve (times of occurrence of the absolute maximum and minimum) were calculated. Cosinor analysis was also applied to weekly (seven 1-day intervals), and monthly (12 1-month intervals), for weekly and monthly analysis, respectively. Significance levels were set at *p* < 0.05.

## Results

A total of 665 patients (264 males, 39.7%) with a mean age of 65.8 ± 8.3 years were included in the study. A precipitating emotional stressor was identified in 176 patients (26.5%), a physical stressor in 196 patients (29.5%). Onset of TGA was known in 646 patients (97.1%). Details of the Mannheim (*n* = 404 patients) and Kansai cohorts (*n* = 261 patients), which did not differ in basic demographic features, are presented in Table 1.

**Table 1:** Basic demographics, frequency of precipitating stressors and TGA occurrence on a weekday (Monday–Friday) in the Mannheim and Kansai cohorts

Variable	Mannheim (*n* = 404)	Kansai (*n* = 261)	*p* value
Age, mean (SD)	66.11 (8.01)	65.31 (8.63)	0.225
Male, *N* (%)	162 (40.1%)	102 (39.1%)	0.793
Trigger emotional, *N* (%)^a^	123 (50.6%)	53 (41.1%)	0.080
Trigger physical, *N* (%)^a^	120 (49.4%)	76 (58.9%)	
TGA on weekday, *N* (%)	299 (74.0%)	164 (62.8%)	**0.002**

^a^Percentages refer to group of patients with a known trigger

### Circadian variation in TGA occurrence

**Fig. 1:**
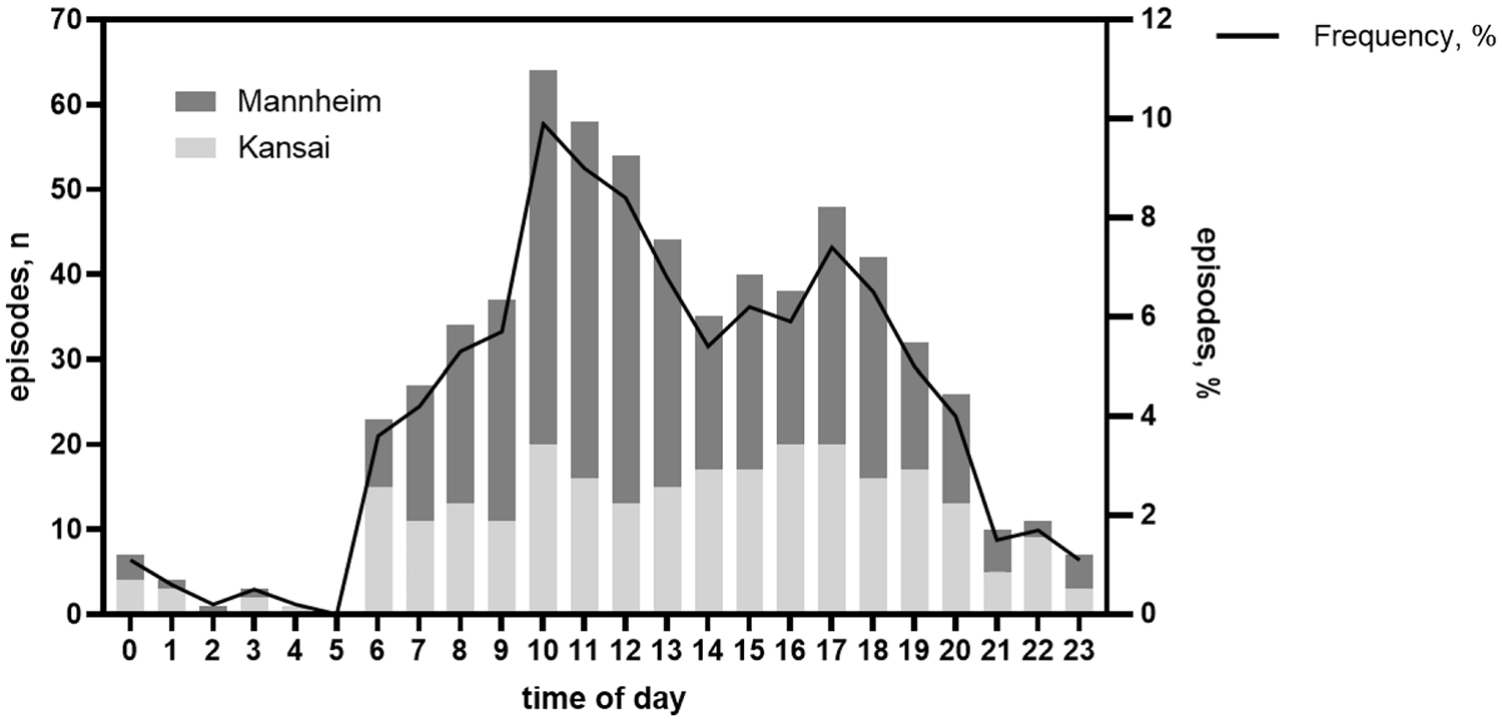
Time-of-day distribution in our cohort of 646 transient global amnesia cases from Japan (Kansai district) and Germany (Mannheim) with known time of onset

A significant circadian rhythm was identified for the entire study population (*p* = 0.002) with a major peak in the morning between 10 and 11 am and a secondary peak between 4 and 5 pm with a trough between 4 and 5 am (Fig. 1). Such a pattern was found regardless of sex (women: *p* = 0.004, main peak between 10 and 11 am, secondary peak non-significant, trough between 4 and 5 am; men: *p* = 0.004, main peak between 9 and 10 am, secondary peak between 4 and 5 pm, trough between 3 and 4 pm) and age (≥ 65 years: *p* = 0.003, main peak between 10 and 11 am, secondary peak non-significant, trough between 4 and 5 am; < 65 years: *p* = 0.0009, main peak between 10 and 11 am, secondary peak between 4 and 5 pm, trough between 4 and 5 am). Moreover, in both German and Japanese sub-cohorts, similar significant circadian rhythms for onset of TGA attacks were found (Mannheim: *p* = 0.003, main peak between 10 and 11 am, secondary peak non-significant, trough between 4 and 5 am; Kansai: *p* = 0.007; main peak between 9 and 10 am, secondary peak between 5 and 6 pm, trough between 3 and 4 am).

An analysis of TGA occurrence in four 6-h intervals did not render any significant results (*p* = 0.245).

### Weekly and weekday/weekend TGA occurrences

**Fig. 2:**
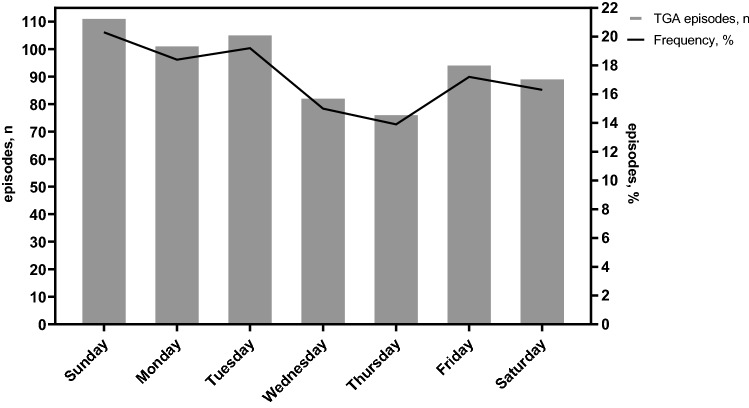
Distribution according to day of the week in our cohort of 665 transient global amnesia cases from Japan (Kansai district) and Germany (Mannheim)

Regarding the variation of TGA occurrence by day of the week, there was no evidence of significant rhythmicity, although a trend was shown for main peak on Sunday (*n* = 112, 20.3% of cases, *p* = 0.087), see Fig. 2. Considering weekday (Monday–Friday) vs. weekend (Saturday/Sunday) occurrence of TGA, there was a significant difference between the Mannheim and Kansai cohorts: the proportion of patients with TGA occurring on a weekday was significantly higher in the Mannheim than in the Kansai cohort (*n* = 299, 74.0% vs. *n* = 164, 62.8%, respectively, *p* = 0.002).

### Monthly and seasonal variation

**Fig. 3:**
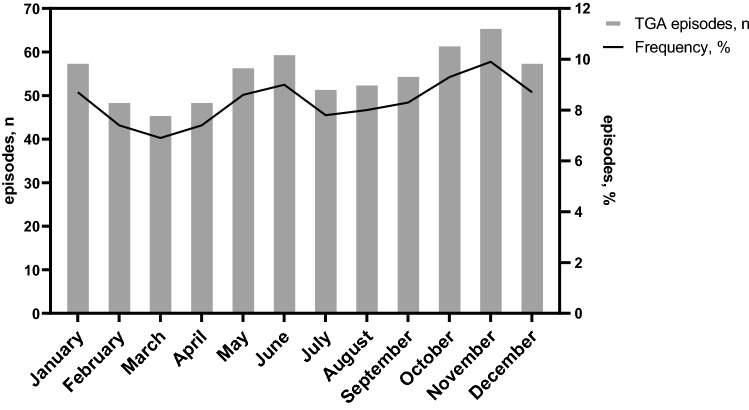
Monthly distribution in our cohort of 665 transient global amnesia cases from Japan (Kansai district) and Germany (Mannheim)

We did not identify any significant monthly (*p* = 0.114) or seasonal (*p* = 0.793) variations in TGA occurrence (Fig. 3).

## Discussion

Our data from two large, well-characterized cohorts of TGA patients reveal the presence of a robust circadian rhythmicity, characterized by a main peak in the morning, an accessory secondary peak in the afternoon, and a trough during the early morning hours. Remarkably, the circadian rhythmicity is found not only in the total sample but for either of the two study sites located on different continents, and is independent of sex and age.

Apparent clustering of TGA attacks in the morning has been repeatedly noted [6, 30–34] but, to the best of our knowledge, we present the first systematic study and formal analysis. The results of our study raise the question how the evident circadian rhythmicity fits within a conceptualization of TGA as a stress-induced condition. The sympatho-adrenomedullary (SAM) and the HPA axes are the major structural–functional correlates of the stress response system with the HPA axis, in particular, employing circadian rhythmicity of secretion to adapt tissue responsiveness to differing demands throughout the day. In addition, ultradian rhythms enable fast reactivity of the HPA system when needed [35]. Normally, the secretion of cortisol, which subserves anticipation, follows a circadian pattern with peak levels shortly before waking and a trough during sleep or prolonged inactivity. Under conditions of acute or chronic stress, however, this pattern is distinctly altered with, for example, constantly elevated cortisol levels during acute stress in the context of cardiac surgery, or disturbance of cortisol pulsatility under conditions of chronic stress [28, 36, 37]. As a consequence, system homeostasis is critically challenged. In this regard, chronobiology may provide one mediating link with respect to the association of TGA with acute or chronic stressors. In addition, significant changes in sympathetic-parasympathetic balance and HPA axis may also be influenced by individual differences in terms of diurnal preferences (the so called ‘chronotype’) and rhythm desynchronizations, e.g., due to shift work, jet lag, and daylight saving time [38].

Another aspect to be considered concerns the relationship between circadian rhythm and cognition, more precisely, memory. It has long been recognized that memory is influenced by circadian rhythms across phyla [39]. In humans, for example, shift work or jet lag, which disturb circadian rhythms, cause cognitive deficits, induce morphological changes within the temporal lobe and impact on hippocampal neurogenesis [40–42] Furthermore, circadian rhythm disruption may increase the risk for neurodegenerative disorders such as Alzheimer’s disease where cognitive deficits and sleep/wake abnormalities are clinically prominent [43, 44].

Memory appears to be differentially influenced or regulated by the circadian system with hippocampus-dependent processes emerging as more markedly impacted on than hippocampus-independent ones [45–47]. The function of the interrelation between circadian rhythms and memory—evolutionarily maintained through the process of natural selection—can only be hypothesized on. It has been suggested that cyclic-dynamic patterns of hippocampal gene expression form a temporal framework for memory formation [48]. Circadian variation in memory performance may also be related to, for example, the conservation of energy during certain phases as memory formation is a metabolically expensive process [49] that, interestingly, has been demonstrated to decrease stress resistance in Drosophila [50]. From this perspective, then, one may speculate that TGA is the result of an individual’s unique signature of traits and/or comorbidities interacting with a complex scenario of confluencing circumstances and conditions which thus confer the chronorisk of the condition. In light of a recent pathophysiological hypothesis for TGA postulating a role for the renin–angiotensin system, specifically Angiotensin II (AT-II) and interactions of central AT-II type 1 and N-methyl-d-aspartate receptors [1] it bears mentioning that plasma levels of AT-II peak mid-morning [51], which coincides with the major peak of TGA occurrence.

Bimodal acrophase patterns, characterized by different peaks and troughs, have also been found for other disorders, such as ischemic and hemorrhagic stroke or acute cardiovascular disorders [52, 53]. Regarding Takotsubo syndrome, which strikingly resembles TGA concerning the stress-related triggers [54–56], a morning preference has been observed as well [57].

We did not observe any type of infradian rhythmicity of TGA occurrence. First, there was no evidence of a significant weekly pattern, although there was a trend toward a higher frequency of events on Sunday. Monthly and seasonal variation have been previously reported in samples from Israel and South Korea [58, 59], both of these studies noted a preference for TGA occurring in the winter, corroborating an observation by Akawi and colleagues [60] and arguing for an association of TGA occurrence with lower ambient temperatures. Other studies, however, found either even monthly and seasonal distributions [61], or identified a summer preference [34, 62, 63]. Hence, the resulting picture is inconclusive, currently not allowing for any definite implications. While the data regarding circadian variation are very robust between the countries, the absence of infradian types of rhythm may be genuine but may also be related to climate differences as well as those related to culture or lifestyle, which all may impact on, for example, weekday vs. weekend and seasonal scales.

In sum, chronobiological investigation of TGA, with particular emphasis on circadian rhythmicity, may provide an additional perspective from which one may approach to solve the puzzle related to the pathomechanism underlying this fascinating condition.

## Data Availability

We are willing to share anonymized data upon request from any qualified investigator.
